# An equation of state for insect swarms

**DOI:** 10.1038/s41598-021-83303-z

**Published:** 2021-02-12

**Authors:** Michael Sinhuber, Kasper van der Vaart, Yenchia Feng, Andrew M. Reynolds, Nicholas T. Ouellette

**Affiliations:** 1 Department of Civil and Environmental Engineering, Stanford University, Stanford, CA 94305 USA; 2 Rothamsted Research, Harpenden, Hertfordshire, AL5 2JQ UK; 3 Present Address: Carl Von Ossietzky Universität Oldenburg, 26129 Oldenburg, Germany

**Keywords:** Biophysics, Behavioural ecology, Biological physics

## Abstract

Collective behaviour in flocks, crowds, and swarms occurs throughout the biological world. Animal groups are generally assumed to be evolutionarily adapted to robustly achieve particular functions, so there is widespread interest in exploiting collective behaviour for bio-inspired engineering. However, this requires understanding the precise properties and function of groups, which remains a challenge. Here, we demonstrate that collective groups can be described in a thermodynamic framework. We define an appropriate set of state variables and extract an equation of state for laboratory midge swarms. We then drive swarms through “thermodynamic” cycles via external stimuli, and show that our equation of state holds throughout. Our findings demonstrate a new way of precisely quantifying the nature of collective groups and provide a cornerstone for potential future engineering design.

## Introduction

Organisms on every size scale, from single-celled^1^ to highly complex^2^, regularly come together in groups. In many cases, such aggregations are collective, in that the group as a whole displays properties and functionality distinct from those of its individual members or simply their linear sum^3,4^*.* It is generally assumed that since evolution has led so many different kinds of animals to behave collectively, the performance of collective groups at whatever task they seek to achieve ought to be well beyond the capabilities of a single individual^5^, while also being robust to uncertain natural environments^6,7^ and operating without the need for top-down control^8^. For these reasons, there has been significant interest both in understanding how collectivity conveys these advantages^9^ and how to exploit it in engineered systems^10,11^.

Taking advantage of evolutionary adaptation for the design of such a bio-inspired artificial collective system requires both determining the interaction rules used by real animals and properly understanding the function of the group. Both of these tasks remain a challenge. Extracting interaction rules by observing group behaviour is a highly nontrivial inverse problem^12^ that can typically only be solved by assuming a modelling framework a priori^13,14^. Appropriate model selection is made more difficult given that interactions may change in different contexts^7,8,15^. Even less work has been done to precisely determine the tasks optimized by collective behaviour. Assumptions about the purpose of group behaviour typically come from ecological reasoning^16^ rather than quantitative empirical evidence^8^—and in some cases, such as hypothesized aerodynamic benefits conveyed to flocking birds, such reasoning has proved to be incorrect^17,18^.

We argue that the essential nature of the group functionality is encoded in its properties—and therefore that understanding these properties both allows one to quantify the purpose of the collective behaviour and to predict the response of the group to environmental changes. As recent work has demonstrated^19–21^, a powerful way to characterize these properties is to borrow ideas from other areas of physics. For groups on the move such as human crowds, hydrodynamics is a natural choice, and empirically measured constitutive laws have allowed the formulation of equations of motion that accurately predict how crowds flow^20^. But for stationary groups such as insect swarms, where the group as a whole does not move even though its constituent individuals are continuously rearranging, thermodynamics is a more natural framework, as it allows one to precisely describe the state of the system irrespective of its net motion^22^. The most fundamental relationship for doing so is the equation of state, which links the state variables that describe the macroscopic properties of the system and encodes how they co-vary in response to environmental changes.

**Figure 1:**
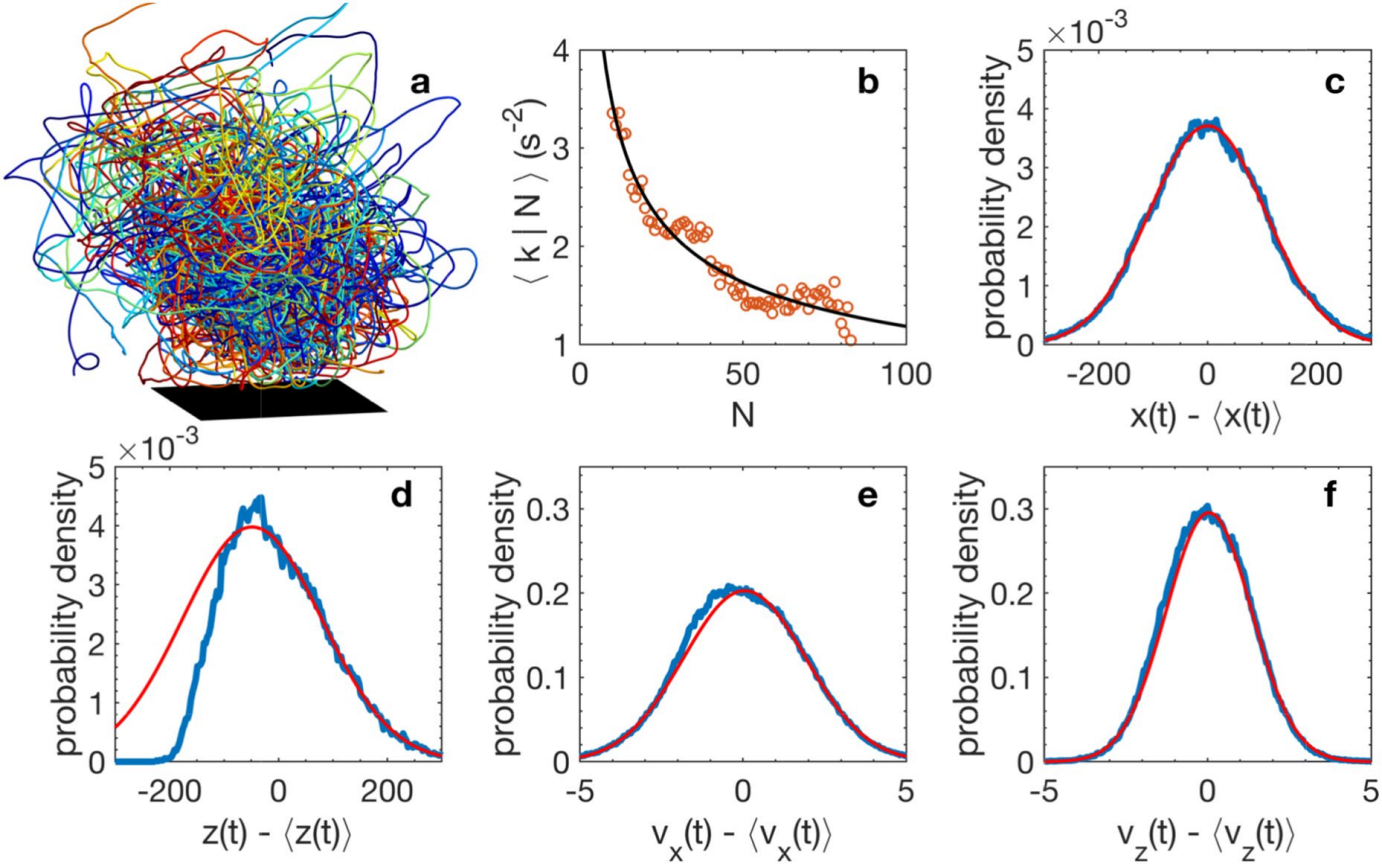
Swarm kinematics. (**a**) Trajectories (> 40 s long) of individual midges (each colour corresponding to a different midge) are individually convoluted but remain spatially localized over a ground-based swarm marker (black square). (**b**) Averaged spring constant $\langle k|N\rangle$ as function of the swarm size *N* (symbols). The black line is a power-law fit to the data. (**c**) Probability density function (PDF) of midge positions in the horizontal plane (blue) along with a Gaussian fit to the data (red). (**d**) PDF of midge positions in the vertical (gravity) direction (blue) and a Gaussian fit to the data (red). The deviation from Gaussianity in the vertical component of the position arises from the symmetry breaking due to the bottom floor of the experimental setup. (**e**,**f**) PDFs of the horizontal (**e**) and vertical (**f**) midge velocities (blue) along with Gaussian fits to the data (red).

Here, we formulate such an equation of state for laboratory swarms of the non-biting midge *Chironomus riparius* (Fig. 1a). We define appropriate state variables, and empirically deduce their relationship by analysing a large data set of measured swarms^23^. Then, by applying a suitable sequence of external perturbations to the swarms, we show that we can drive them through a thermodynamic cycle in pressure–volume space throughout which our empirical equation of state holds.

## Results

### State variables

The first step in describing the macroscopic properties of the swarm is to define a set of state variables that fully characterizes the state of the system. The equation of state then links these state variables in a functional relation. In classical thermodynamics, a complete set of state variables is given by the conjugate pairs of pressure *P* and volume *V*, temperature *T* and entropy *S*, and, if the number of particles is not fixed, chemical potential *μ* and number of particles *N*. We use an analogous set of state variables here to characterize swarms. The most straightforward state variable to define is the number of individuals *N*, which is given simply by the number of midges in the swarm at a given time (note that midges that are *not* swarming simply sit on the walls or floor of the laboratory enclosure). The volume *V* of the swarm can be straightforwardly defined and computed as the volume of the convex hull enclosing all the midges. Note that while *N* and *V* are not independently controllable quantities, the ratio *N*/*V* is empirically approximately constant in large swarms^24^, meaning that the “thermodynamic” limit (that is, $N \to \infty$ and $V \to \infty$ with $\frac{N}{V} \to \rho$) is approached in our swarms^25^. In typical swarming events, *N* changes on a time scale that is very slow compared to the swarm dynamics; thus, a chemical potential is not needed to describe the instantaneous state of the swarm. Note, though, that since the number of midges varies between measurements that may be separated by many days, *N* remains a relevant state variable for capturing swarm-to-swarm variability.

The remaining three state variables are somewhat more subtle, but can be defined by building on previous work. It has been explicitly shown^26^ that a virial relation based on the kinetic energy and an effective potential energy holds for laboratory swarms of *Chironomus riparius*. For particles moving in a potential, this virial relation can be used to define a pressure^26^. As we have shown previously, swarming midges behave as if they are trapped in a harmonic potential well that binds them to the swarm, with a spring constant *k*(*N*) that depends on the swarm size^24,26^ (Fig. 1b). The difference between the kinetic energy and this harmonic potential energy thus allows us to compute a pressure^4,26,27^, which is conceptually similar to the swim pressure defined in other active systems^28^. The virial theorem thus provides a link between kinetic energy, potential energy, and a field that plays the role of a pressure, when coupled with the observation that individual midges to a good approximation behave as if they are moving in a harmonic potential^24,26^. We can write this virial pressure *P* (per unit mass, assuming a constant mass per midge) as

$$P = \frac{1}{3NV}\mathop \sum \limits_{i = 1}^{N} \left( {v_{i}^{2} - \frac{1}{2}\langle k\rangle r_{i}^{2} } \right),$$

where *N* is the number of midges in the swarm, *V* is the swarm volume, *v*_i_ is the velocity of midge *i*, *r*_i_ is its distance from the swarm centre of mass, and $\langle k\rangle = \langle - {\mathbf{a}}_{i} \cdot \hat{\mathbf{r}}_{i} / r_{i} \rangle$ is the effective spring constant of the emergent potential well that binds midges to the swarm. In this expression, **a**_i_ is the acceleration of midge *i*, $\hat{\mathbf{r}}_{i}$ is the unit vector pointing from a midge towards the instantaneous centre of mass of the swarm (defined as $1/N\sum\nolimits_{i = 1}^{N} {{\mathbf{r}}_i }$) and averages are taken over the individuals in the swarm. This spring constant depends on the swarm size *N* (Fig. 1b). We note that we have previously simply used the directly computed potential energy $- \langle {\mathbf{a}}_{i} \cdot {\mathbf{r}}_{i} \rangle$ to define the pressure^4,27^; here, we instead average the potential terms and fit them to a power law in *N* (Fig. 1b) to mitigate the contribution of spurious instantaneous noise in the individual positions that would be enhanced by differentiating them twice to compute accelerations. We use this power law to determine the spring constant *k* instantaneously at each time step.

The results from the two methods for computing the pressure are similar and consistent, but the method we use here is less prone to noise. Physically, this pressure *P* can be interpreted as the additional spatially variable energy density required to keep the midges bound to the swarm given that their potential energy varies in space but their mean velocity (and therefore kinetic energy) does not. Thus, compared to a simple passive particle moving in a harmonic well, midges have more kinetic energy than expected at the swarm edges; this pressure compensates for the excess kinetic energy. This pressure should be viewed as a manifestation of the active nature of the midges (similar to a swim pressure^28^), since the kinetic energy is an active property of each individual midge and the potential energy is an emergent property of the swarm.

We can define a Shannon-like entropy *S* via its definition in terms of the joint probability distributions of position and velocity. This entropy is defined as

$$S = - \mathop \smallint \limits_{ - \infty }^{\infty } p(x,\;v)\log_{2} p(x,\;v)dxdv,$$

where *p*(*x*,*v*) is the joint probability density function (PDF) of midge position and velocity. *S* here is measured in bits, as it is naturally an information entropy. Empirically, we find that the position and velocity PDFs are nearly statistically independent for all components and close to Gaussian, aside from the vertical component of the position (Fig. 1c–f). However, the deviation from Gaussianity in this component (which occurs because of the symmetry breaking due to the ground) does not significantly affect the estimate of the entropy; thus, we approximate it as Gaussian as well. Making these approximations, we can thus analytically write the (extensive) entropy as

$$S = \frac{3N}{{\ln 2}}\ln \left( {2\pi e\sigma_{x} \sigma_{v} } \right),$$

where $\sigma_{x}$ and $\sigma_{v}$ are the standard deviations of the midge positions and velocities, respectively. In practice, we calculated $\sigma_{v}$ by averaging the instantaneous root-mean-square values of all three velocity components rather than a time-averaged value; the difference between these components was always less than 10%. This expression makes it more clear why the Gaussian approximation for the vertical component of the position is reasonable here: only the mean and variance of the PDFs are required to compute the entropy, and these low moments are very similar for the true data and the Gaussian estimate.

Although there is no obvious definition of temperature for a swarm, we can define one starting from the entropy, since temperature (when scaled by a Boltzmann constant) can be defined as the increase in the total physical energy of the system due to the addition of a single bit of entropy. Given our definitions, adding a single bit of entropy (that is, setting $S \to S + 1$) for constant $\sigma_{x}$ and *N* (that is, a swarm of fixed number and spatial size) is equivalent to setting $\sigma_{v} \to 2^{1/(3N)} \sigma_{v} .$ Adding this entropy changes the total energy of the system by an amount

$$\frac{3}{2}\sigma_{v}^{2} N\left( {2^{\frac{2}{3N}} - 1} \right) \equiv k_{B}^{*} T,$$

which we thus define as the temperature $k_{B}^{*} T$. Even though this temperature is nominally a function of the swarm size *N,* it correctly yields an intensive temperature as expected in the limit of large *N*, as the explicit *N*-dependence vanishes in that limit since $\lim_{n \to \infty } k_{B}^{*} T = \sigma_{v}^{2} \ln 2$. In practice, this limit is achieved very rapidly: we find that this temperature is nearly independent of *N* for *N* larger than about 20, consistent with our earlier results on the effective “thermodynamic limit” for swarms^25^. The effective Boltzmann constant $k_{B}^{*}$ is included here to convert between temperature and energy, though we note that we cannot set its value, as there is no intrinsically preferred temperature scale.

### Equipartition

**Figure 2:**
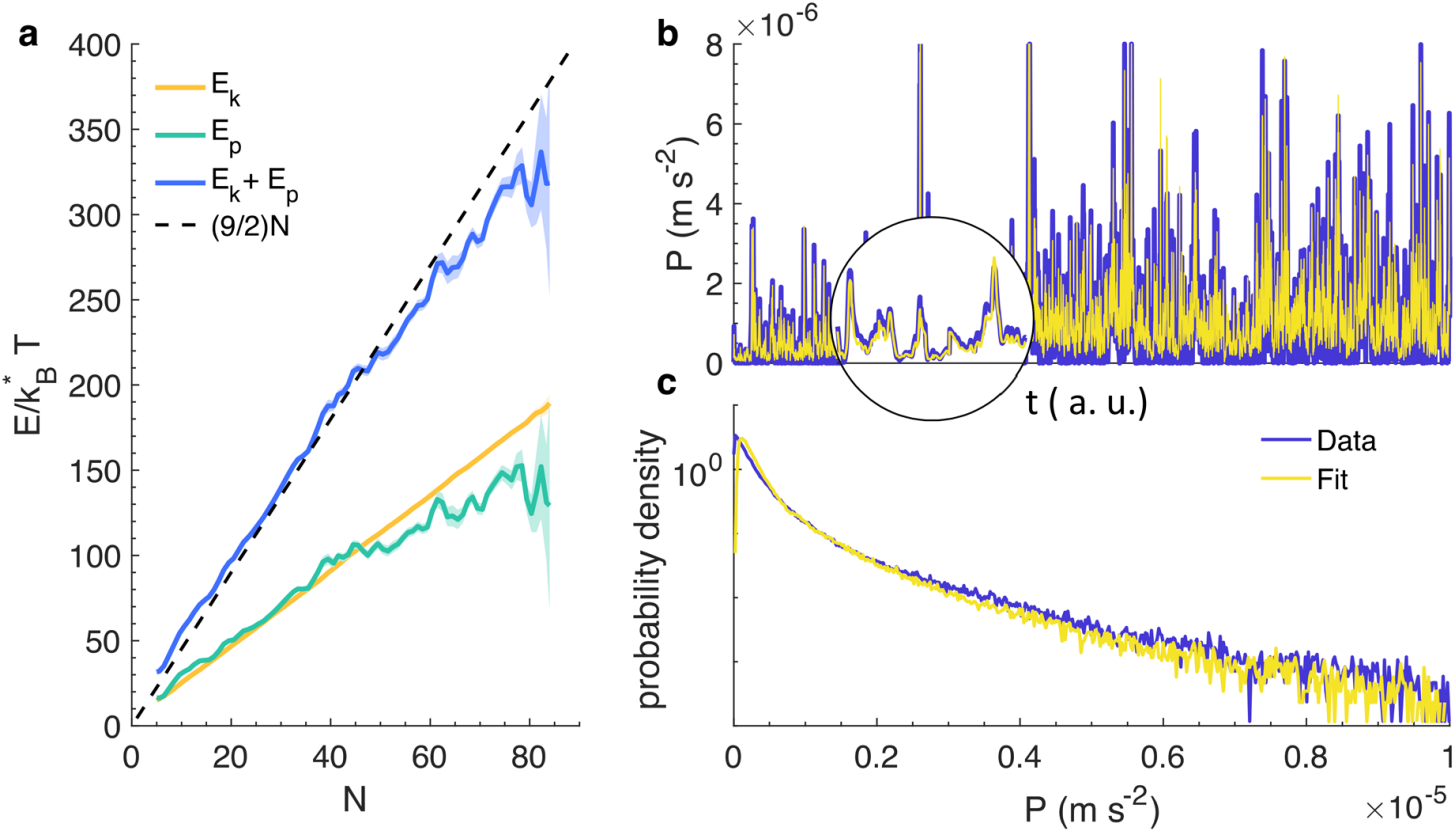
Equipartition and the equation of state. (**a**) The total energy of the system $E$ normalized by $k_{B}^{*} T$ as a function of swarm size (blue) along with the kinetic energy $E_{k}$ (yellow) and potential energy $E_{p}$ (blue). The total normalized energy of the system is well approximated by (9/2)*N* (black dashed line), indicating that each individual midge contributes $(9/2)k_{B}^{*} T$ to *E* and thus has 9 degrees of freedom (6 after discounting the factor of ${\text{ln}}2$ in our definition of $k_{B}^{*} T$). The deviations from that behaviour for the largest swarms can be attributed to a growing uncertainty in the energy due to the smaller number of experiments with such large swarms. (**b**) A portion of our ensemble of data of the measured pressure (blue). The yellow line is the reconstruction of the pressure from our equation of state. The inset shows a zoomed-in portion of the data to highlight the quality of the reconstruction. (**c**) PDF of the pressure for our entire data ensemble^23^. The statistics of the directly measured pressure (blue) and reconstructed pressure from the equation of state have nearly identical statistics for the full dynamic range of the signal.

With these definitions in hand, we can evaluate the suitability of these quantities for describing the macroscopic state of midge swarms. First, we note that proper state variables ought to be independent of the swarm history; that is, they ought to describe only the current state of the system rather than the protocol by which that state was prepared. Although this property is difficult to prove incontrovertibly, none of the definitions of our state variables have history dependence. We further find that when these state variables are modulated (see below), their correlation times are very short, lending support to their interpretation as true state variables. We can also compare the relationships between these state variables and the swarm behaviour to what would be expected classically. In equilibrium thermodynamics, for example, temperature is connected to the number of degrees of freedom (d.o.f.) in a system via equipartition, such that each d.o.f. contributes an energy of $\frac{1}{2}k_{B}^{*} T$. We can write the total energy *E* of a swarm as the sum of the kinetic energy $E_{k} (t) = \frac{1}{2}v^{2}$ and potential energy $E_{p} (t) = \frac{1}{2}k(N)r(t)^{2}$ for all the individuals, where *r* is the distance of a midge to the swarm centre of mass, *v* is the velocity of a midge, and *k*(*N*) is the effective spring constant. Surprisingly, even though individual midges are certainly not in equilibrium due to their active nature, we find that the total energy is linear in both *T* and *N* (Fig. 2a), and that there is no apparent anisotropy, suggesting that equipartition holds for our swarms. This result is highly nontrivial, especially given that our definition of *T* does not contain the spring constant *k*(*N*), which is only determined empirically from our data. Moreover, the slope of the $E/k_{B}^{*} T$ curve is well approximated as (9/2)*N*, implying that each midge has 9 effective d.o.f. (or 6 after discounting the factor of ${\text{ln}}2$ in our definition of $k_{B}^{*} T$) These d.o.f. can be identified as 3 translational and 3 potential modes, given that the potential well in which the midges reside is three-dimensional. These results demonstrate the surprising applicability of equilibrium thermodynamics for describing the macroscopic state of swarms^29^.

### Equation of state

The fundamental relation in any thermodynamic system is the equation of state that expresses how the state variables co-vary. Equations of state are thus the foundation for the design and control of thermodynamic systems, because they describe how the system will respond when a subset of the state variables are modulated. Any equation of state can be written in the form $P = f(V, \;T,\;N)$ for some function *f.* Although the form of *f* is a priori unknown, it can typically be written as a power series in *V*, *T*, and *N*, in the spirit of a virial expansion. We fit the equation of state to our data assuming the functional form

$$P = f(V, \;k_{B}^{*} T,\;N) = c_{4} V^{{c_{1} }} (k_{B}^{*} T)^{{c_{2} }} N^{{c_{3} }},$$

and using nonlinear least-squares regression. We chose to fit to the pressure for convenient analogy with a thermodynamic framework, but any other variable would have been an equivalent possibility. We note that when fitting, we normalized all the state variables by their root-mean-square values so that they were all of the same order of magnitude. These normalization pre-factors do not change the exponents, but are instead simply absorbed into $c_{4}$. Thus, to leading order, we assume $P = f(V,\;k_{B}^{*} T,\;N) \propto V^{{c_{1} }} (k_{B}^{*} T)^{{c_{2} }} N^{{c_{3} }}$ and fit this relation to the swarm pressure (Fig. 2b,c), obtaining *c*_1_ = − 1.7, *c*_2_ = 2, and *c*_3_ = 1, with uncertainties on the order of 1%. Although the expression for the pressure does depend on three parameters in a nonlinear fashion, the resulting estimates for these parameters are remarkably stable and consistent across all measurements. Hence, we arrive at the equation of state $PV^{1.7} \propto N(k_{B}^{*} T)^{2} .$

This equation of state reveals aspects of the nature of swarms, particularly when compared with the linear equation of state for an ideal gas (where $PV = Nk_{B} T$). In both cases, for example, to maintain a fixed pressure and volume, smaller systems need to be hotter; but this requirement is less severe for swarms since the temperature is squared, meaning that midges have to speed up less than ideal gas molecules do. Likewise, to maintain a fixed temperature, volume expansion must be counteracted by a reduction in pressure; but midges must lower the pressure more than a corresponding ideal gas, which is reflective of the decrease of the swarm spring constant with size.

### Thermodynamic cycling

**Figure 3:**
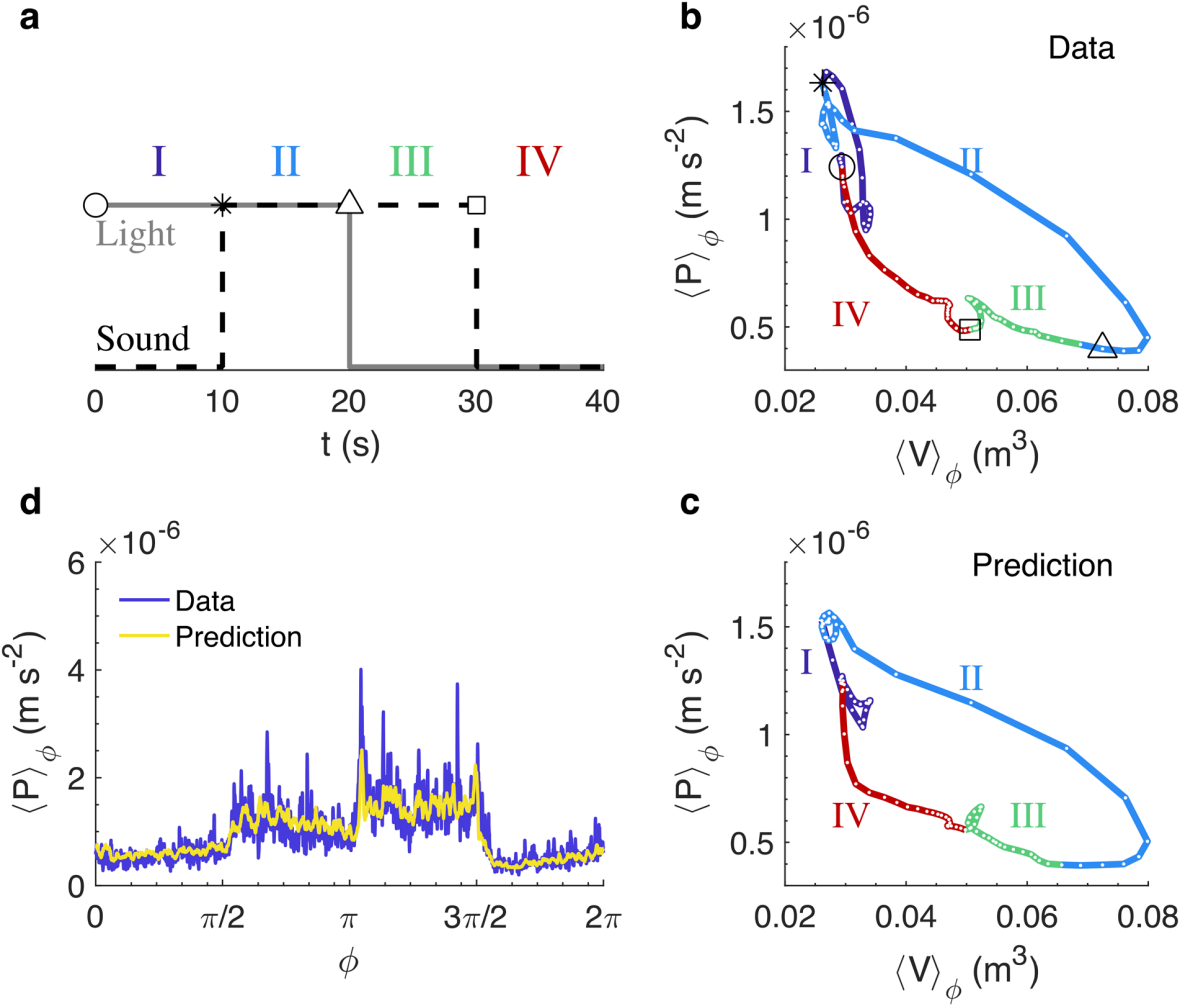
Thermodynamic cycling of a midge swarm with $\langle N\rangle = 27$. Schematic of the perturbation cycle showing the illumination (solid) and sound (dashed) signal timings. The symbols indicate the switching points identified in (**b**). (**b**,**c**) Phase-averaged swarm behaviour during the perturbation cycle plotted in the pressure–volume phase plane for (**b**) the pressure signal as measured and (**c**) as reconstructed using our equation of state. $\left\langle {\,} \right\rangle_{\phi }$ denotes a phase average of a quantity over a full cycle. The four different states of the perturbation signal are indicated. The data has been averaged using a moving 3.5-s window for clarity. The swarm behaviour moves in a closed loop in this phase plane during this cycling, as would be expected for an engine in equilibrium thermodynamics, and the equation of state holds throughout even though it was developed only for unperturbed swarms. (**d**) Phase-averaged pressure $\langle P\rangle_{\phi }$ of the swarm during a continuous cycle through the four light and sound states. The blue line shows the directly measured pressure and the yellow line shows the reconstruction using the equation of state.

Beyond such reasoning, however, the true power of an equation of state in thermodynamics lies in specifying how the state variables will change when some are varied but the system remains in the same state, such as in an engine. To demonstrate that our equation of state similarly describes swarms, it is thus necessary to drive them away from their natural state. Although it is impossible to manipulate the state variables directly in this system of living organisms as one would do with a mechanical system, we have shown previously that time-varying acoustic^30^ and illumination^27^ stimulation lead to macroscopic changes in swarm behaviour. Here we therefore build on these findings and use interlaced illumination changes and acoustic signals to drive swarms along four distinct paths in pressure–volume space, analogous to a thermodynamic engine cycle. The stimulation protocol is sketched in Fig. 3a. The “on” state of the acoustic signal is telegraph noise (see Experimental details), while the “off” state is completely quiet. The illumination signal simply switches between two different steady light levels. Switching between the four states of “light-high and sound-on,” “light-high and sound-off,” “light-low and sound-off,” and “light-low and sound-on” with a 40-s period (Fig. 3a) produces the pressure–volume cycle shown in Fig. 3b. We suspect that the loops in the cycle stem from the swarm’s typical “startle” response after abrupt changes in environmental conditions, followed by a rapid relaxation to a steady state^27,30^.

In addition to the pressure and volume, we can also measure the other state variables as we perturb the swarms. Given that we do not observe any evidence of a phase transition, we would expect that our equation of state, if valid, should hold throughout this cycle. To check this hypothesis, we used the measured *V*, *T*, and *N* values during unperturbed experiments along with the equation of state to predict the scaling exponents, and in turn the pressure *P*. We then use these baseline, unperturbed exponents and *V*, *T*, and *N* during the interlaced perturbations to predict a pressure *P*. This pressure prediction matches the measured signal exceptionally well (Fig. 3c,d) even though the equation of state was formulated only using data from unperturbed swarms, highlighting the quality of this thermodynamic analogy. Although we might expect that a strong enough perturbation might lead to qualitatively different behaviour (if the swarm went through the analog of a phase transition^31^), our results give strong support to the hypothesis that our equation of state should hold for any perturbation that does not drive such a transition.

## Discussion

Our findings demonstrate the surprising efficacy of classical equilibrium thermodynamics for quantitatively characterizing and predicting collective behaviour in biology. Even though individual midges are certainly not in equilibrium and need not obey the same rules as, for example, particles in an ideal gas, we find that the collective behaviour of ensembles of these individuals is surprisingly simple. The existence of a well-defined equation of state for this system gives us a new way both of illuminating the purpose of collective behaviour, given that it encodes the nature of the collective state, and quantitatively distinguishing different kinds of animal groups that may have similar movement patterns but different functions^1–3,8^. Importantly, we note that this equation of state is not a swarm model per se, in that it does not make any detailed predictions about the dynamics of individuals. Rather, it gives us a quantitative way of analysing and interpreting swarm data at the macroscale. Finally, these results also provide a natural starting point for designing artificial collective systems by outlining a framework for adapting intuition and expertise gained from engineering thermodynamic systems to this new situation. This approach could, for example, be useful to guide the design of engineered drone swarms via machine learning techniques^32^ and to provide a precise and quantifiable global description of the collective nature of swarms.

## Methods

In our laboratory we maintain a colony of *C. riparius* midges in an (122 cm)^3^ acrylic tank. *C. riparius* larvae develop in eight 10 L breeding tanks filled with dechlorinated, aerated water and a cellulose substrate. The colony is regulated on an artificial circadian rhythm with 16 h of light and 8 h of night using an overhead light on a timer. Over the roughly 2-week life cycle of the midges, larvae become pupae and eventually mature into flying adult midges. Females in the colony mate with males, fertilizing eggs that they lay in the breeding tanks, thus closing the life cycle.

Just after dusk and dawn, male midges will form mating swarms over ground-based visual features known as swarm markers^33^. In our laboratory, this feature is a black square plate. Swarms are consistently spheroidal with a swarm diameter that depends on the number of swarming individuals^24^. Typical swarm sizes in our laboratory range from 10 to 100 individuals. Note that individuals that are not participating in the swarm do not fly; rather, they sit on the walls or floor of the enclosure. The swarm behaviour is recorded by three cameras placed outside the enclosure.

The cameras used to image the swarms were hardware-synchronized Point Grey Flea3 1.3 MP Mono USB3 Vision cameras running at 100 frames per second, synchronized via an external function generator. To illuminate the midges without interfering with their natural behaviour, we used 20 3 W near-infrared LED arrays placed on top of and inside the measurement tank. *C. riparius* do not see in the infrared, but it is detectable by the cameras, thus allowing non-intrusive imaging of the swarming events. The cameras were placed on tripods outside the midge enclosure, and were arranged in a horizontal plane with angular separations of 30° and 70°^23^ and placed far enough from the experimental enclosure to ensure that the full swarm was always fully within the field of view of each camera. Calibration of the imaging system was done via Tsai’s method^34^, using a flat plate with a regular dot pattern placed inside the tank (and removed before the initiation of swarming) as a calibration reference. During each acquisition session, each of which typically occurred on different days, we recorded between 30,000 and 100,000 frames of data, corresponding to 5–16 min and 40 s of swarming. To obtain three-dimensional trajectories from the individual camera recordings, we first processed each image to obtain 2D midge positions in each camera’s frame of reference, matched the data between the cameras to obtain 3D midge positions for every midge in the swarm, and finally tracked all the 3D positions in time. The observed swarms are dilute. Even in statistically unusual cases of close midge encounters, individuals can still be identified^23^. To process the images, we first removed the background illumination field (obtained by averaging over the full image sequence) and then detected midges simply by computing the centroids of connected regions that were brighter than an empirically set threshold and larger than a minimal pixel size. Regions that were highly non-spherical and very large indicated the overlap of the images of multiple midges in the camera’s field of view, and so were split into multiple midges (see Ref.^23^). The 2D midge coordinates were stereo-matched between the cameras by projecting the lines of sight connecting each camera’s centre of projection and each midge’s 2D location into 3D space using the calibrated camera model and then identifying near-intersections. In principle, two cameras are sufficient for this purpose, but additional cameras have been shown to significantly improve the confidence and yield of this procedure^35^. To connect the 3D positions temporally and create trajectories, we used a multi-frame predictive tracking algorithm^23,35^. Velocities and acceleration were then computed by differentiating the trajectories in time^23^. At each time-step, we additionally removed midges that were sitting or walking on the walls or marker rather than flying, identifying them based on a 100-frame moving average of their speed. If this average speed at a given time step was less than 60 mm/s, we discarded the individual at that time-step.

In this study, we applied interleaved perturbations of two different classes to the swarms in conjunction to the observation of unperturbed swarming events. For the first perturbation type, we induced illumination perturbations, generated by a 6500 K Luxeon Star LED array mounted above the midge enclosure, as described in Ref.^27^. For this study, we modulated the brightness of the LED between 1.4 lux and 2.4 lux, switching every 20 s for a period of 40 s. A second class of perturbations were acoustic signals that were generated by a small (~ 5 cm) omnidirectional speaker placed on the swarm marker. We alternated between a quiet state (that is, no sound played through the speaker) and playback of a telegraph noise acoustic signal, again with a 40-s period. This corresponds to up to 25 full cycles per acquisition session. The telegraph noise was constructed by passing a white-noise signal through a low-pass 700 Hz filter, and then playing short pulses of this signal during the acoustic “on” state with varying length and amplitude. We empirically found that filtering the white-noise signal was necessary to induce a persistent response of the swarm. This may be due to swarms’ tendency to adapt to and ignore static changes in their environment while responding persistently to dynamic changes^27,30^. The pulse length ranged from 0.1 to 0.3 s and the pause between pulses ranged from 0.25 to 0.5 s. The noise amplitude was clearly audible over the ambient sound levels in the laboratory, and we varied it only slightly.

## Data Availability

The trajectory data are available at https://doi.org/10.6084/m9.figshare.11791071.v1.
